# Estimation of the shared mobility demand based on the daily regularity of the urban mobility and the similarity of individual trips

**DOI:** 10.1371/journal.pone.0238143

**Published:** 2020-09-17

**Authors:** Cyril Veve, Nicolas Chiabaut

**Affiliations:** Univ. Lyon, Univ. Gustave Eiffel, ENTPE, LICIT, Lyon, France; The University of Tokyo, JAPAN

## Abstract

Even if shared mobility services are encouraged by transportation policies, they remain underused and inefficient transportation modes because they struggle to find their customer base. This paper aims to estimate the potential demand for such services by focusing on individual trips and determining the number of passengers who perform similar trips. Contrary to existing papers, this study focuses on the demand without assuming any specific shared mobility system. The experiment performed on data coming from New York City conducts to cluster more than 85% of the trips. Consequently, shared mobility services such as ride-sharing can find their customer base and, at a long time, to a significantly reduce the number of cars flowing in the city. After a detailed analysis, commonalities in the clusters are identified: regular patterns from one day to the next exist in shared mobility demand. This regularity makes it possible to anticipate the potential shared mobility demand to help transportation suppliers to optimize their operations.

## Introduction

Recent studies of human behavior reveal the predictability in the mobility of individuals [1, 2]. A significant regularity appears at a larger scale, making it possible to identify recurrent trends in urban mobility even if variability can be observed in the trips at the scale of a particular individual [3] and to derive behavioral laws [4–6]. It appears that patterns repeating from one day to the next can be identified either by observing the traffic conditions [7], the most used roads [8, 9], or in the choice of modes of transport [10]. Despite this conformity in individual human behavior, private cars and taxis (and related services of transportation network companies) remain largely inefficient modes of transport, carrying less than 1.5 passengers on average, leading to significant traffic congestion and generating irreversible economic and environmental impacts [11]. Therefore, there is a significant challenge to massifying transport by encouraging travelers to share either their vehicle or their trip [12]. At least, it necessitates two essential ingredients: the existence of transportation services to match similar travel requests; and the intrinsic potential for ride-sharing of the transportation demand. In practice, this ensures that, every day, shared mobility systems will have enough demand and that any travelers that want to share their trip will find a suitable and efficient solution.

If the literature devoted to the optimization of shared mobility services is large [13–15], it remains significantly weak on the real capacity of an urban network to gather individuals actually making similar routes. However, two recent studies are of particular interest as they quantify the benefit of vehicle pooling for the urban network [16, 17]. Two tremendous results are obtained. Firstly, the analysis of data coming from New York City taxis shows that sharing the ride by two or three customers simultaneously and optimizing the match would lead to a 40% reduction of the total distance traveled by the fleet of taxis [16]. Secondly, an empirical universal law is exhibited, at a macroscopic scale, to qualify the capacity of the users of the transportation network to share their trips [17]. These results are fascinating and encourage us to go even further by trying to group as many passengers as necessary and to focus on the spatio-temporal features of the shareable trips of a city. The particularly interesting point is to determine if we can reveal a day-to-day regularity in the shared mobility demand patterns that can be used, at a long time scale, to design and provide adapted transportation services.

To this end, we try to identify individuals that perform similar trips on an urban network and then construct groups of trips sharing the same spatio-temporal characteristics: origin and destinations whereabouts, departure, and arrival times. Indeed, even if many psychological considerations play a significant role in the transportation mode choice of individuals [18], travel times, and the delays imposed by ride-sharing are determinant. Moreover, we show that these groups of trips exhibit day-to-day regularity, although different individuals realize these trips. Therefore, it seems conceivable that this demand can be realized, in the long term, into the same vehicle. Indeed, the stability of the demand is vital for ride-sourcing / ride-sharing services and individuals that want to use shared transportation modes. An extreme example is the case of the airports where travelers and taxis, respectively, are confident of finding a transportation supply and a patron, respectively. Consequently, identifying day-to-day regular patterns is essential to ensure that shared mobility services can be long-standing propositions to fight against single-occupancy vehicles, reduce the traffic flow, and tend towards a more virtuous mobility [19].

The rest of the paper is organized as follows. Section 1 introduces the methodology used to detect similar trips and estimate demand patterns. Section 2 focuses on the analysis of the results for both scales: daily similar trip clusters and day-to-day mobility patterns. Finally, Section 3 is devoted to a final discussion.

## Estimation of the shared mobility services’ demand

Observing human mobility has become an easier task with the development of telecommunication devices. Individual trips can now be effortlessly tracked through mobile phone data, embedded GPS in vehicles, or many other sources of data [1]. Even if it is now possible to collect all the travelers’ entire trajectories, we only consider origin/destination locations and departure/arrival times of users to represent their trip. There are at least two main reasons. First, such observations can be derived from more numerous sources of data than trajectories (such as origin-destination matrices or other data often aggregated at the transportation analyses zone level). Second, two individuals may have the same origin/destination and departure/arrival times but use different routes, which has no impact on our study. To this end, we use a dataset released by the New York City Taxi and Limousine Commission (https://www1.nyc.gov/site/tlc/index.page) that are freely available. Although these data are not fully representative of human mobility since they only correspond to taxi trips, such a dataset provides an attractive proxy for studying the individuals’ routes within a city. The study focuses on morning peak hours from 8h to 11h and a well known high-density area in terms of mobility in New-York City: Midtown and Upper East Side. For each trip *i*, the dataset gives access to the following information: departure time $t_{i}^{PU}$ and location $p_{i}^{PU}=\left(x_{i}^{PU},y_{i}^{PU}\right)$ of the pick-up of the passenger(s); and arrival time $t_{i}^{DO}$ and location $p_{i}^{DO}=\left(x_{i}^{DO},y_{i}^{DO}\right)$ of the drop-off. All the notations used in the paper are listed in Table 1.

**Table 1:** Nomenclature.

Notation	General
*l*	Pick-up (PU) / Drop-off (DO)
$t_{i}^{PU}$	Departure time of trip i
$p_{i}^{PU}$	PU location of trip i
$t_{i}^{DO}$	Arrival time of trip i
$p_{i}^{DO}$	DO location of trip i
$d(p_{i}^{l},p_{j}^{l})$	Euclidean distance between *l* locations
$|t_{i}^{l}-t_{j}^{l}|$	Difference between *l* times
	**Distance function**
$\bar{S(i,j)}$	Similarity index without penalties
*S*(*i*, *j*)	Similarity index with penalties
*f*^*l*^(*i*, *j*)	Feasibility function
*α*_*l*_	Coefficient to penalize the delay
*γ*	Average pace to connect travelers
$\delta_{x}^{l}$	Spatial threshold
$\delta_{t}^{l}$	Temporal threshold
	**Clustering**
*n*_*k*_	Number of trips in the cluster *k*
*Q*(*k*)	Quality index of the cluster *k*
*Q*_*max*_	Maximal dissimilarity of clusters
$\bar{D_{k}^{l}}$	Average distance between *l* locations in cluster *k*
$\bar{T_{k}^{l}}$	Average offset between *l* times in the cluster *k*
$\bar{l_{k}}$	Average length of *n*_*k*_ trips within cluster *k*
$\bar{\tau_{k}}$	Average duration of *n*_*k*_ trips within cluster *k*
*K*	Number of estimated cluster
*n*	Total number of trips before clustering
$\tilde{n}$	Total number of trips that have been clustered
*ρ*	Ratio of trips clustered $n/\tilde{n}$

The data used in the paper comes from taxi and limousine services. However, the method presented works with any data type, as long as we have access to the origin/destination whereabouts of the trips. Nevertheless, as we can see in [20], taxi trips represent almost a third of total trips in NYC. The authors of this study also underline the fact that a taxi is used, on average 60% of the time. Consequently, these data constitute an excellent indicator to describe the urban mobility of NYC. Because the private car data are not available, it is not easy to precisely estimate the representativeness of taxi trips compared to the total of private car trips, except for this rough value of one-third given by many communications. It is almost sure that taxi users’ are not recurrent users of private cars. Therefore, the demand identified in this study is likely to use the transit system. However, we assume that a significant ratio of taxi trips is comparable to private car trips.

As already stated, the methodology used in this paper to gather observed trips for a given day is based on two successive steps. First, an index of similarity is defined that gives the potentiality between each pair of individual trips to be gathered. Second, using this function, trips are clustered to identify groups of users that can potentially share the same vehicle to travel based on the spatio-temporal features of their trips.

Two different categories of ride-splitting scenarios can be defined: (i) travelers share the entire trip, and (ii) travelers share only a part of the trip. The second category is a source of many interesting studies that mainly focus on the optimization of shared mobility services [13]. On the opposite, our work only considers the first category when users share a vehicle for the entire trip, i.e., multi-hop service is out of the scope.

### Modeling similarity between individual trips

Even if several attempts exist in the literature to express the similarity between two trips [21–23], these studies mainly aim at estimating the likeness between trajectories using variants of Euclidean distance between points of interest or at measuring the number of points shared by two trajectories [24, 25]. This issue is quite different from our goal because the number of observations is much higher in these previous cases. The existing methods to estimate the likeness between trips based on variants of the Longest Common Subsequence Problem (LCSS) need a critical number of observation points to define trips correctly. Here, only the origin and destination whereabouts and times are considered. This choice has been motivated by two main reasons. The first one is the simplicity of our method and the low complexity of calculating the similarity index. The second reason is data accessibility. Many datasets exist for which we do not have access to trajectories to describe trips, but only pairs origin/destination. This method has been developed to estimate the similarity between trips easily and to work with a large number of datasets, not only with datasets containing trajectories. Unfortunately, the research on the similarity between origin and destination of individual trips is very sparse. The main contribution to our work on the similarity between trips is the study of [26]. The authors introduce a similarity measure calculated as the arithmetic mean of the distance between origin/destination locations to evaluate the proximity between two trips *i* and *j*. This index is the weighted geometric mean of Euclidean similarity (spatial) and their temporal similarity. In the following *l* indicates a pick-up or a drop off, $p_{i}^{l}$ and $t_{i}^{l}$ indicate respectively the spatial position and the timestamp of a point *i*. To be consistent with our modeling framework, we can formulate the index as follows:

$$\begin{array}{c} Sim\left(i,j\right)={\left(\frac{1}{2}\sum_{l\in [PU,DO]}e^{\frac{w_{1}\ln\left(\frac{1}{1+d(p_{i}^{l},p_{j}^{l})}\right)+w_{2}\ln\left(\frac{1}{1+|t_{i}^{l}-t_{j}^{l}|}\right)}{w_{1}+w_{2}}}\right)}^{-1} \end{array}$$ (1)

where *d* is the geodesic distance and *w*_1_ and *w*_2_ are weighting factors.

**Fig 1:**
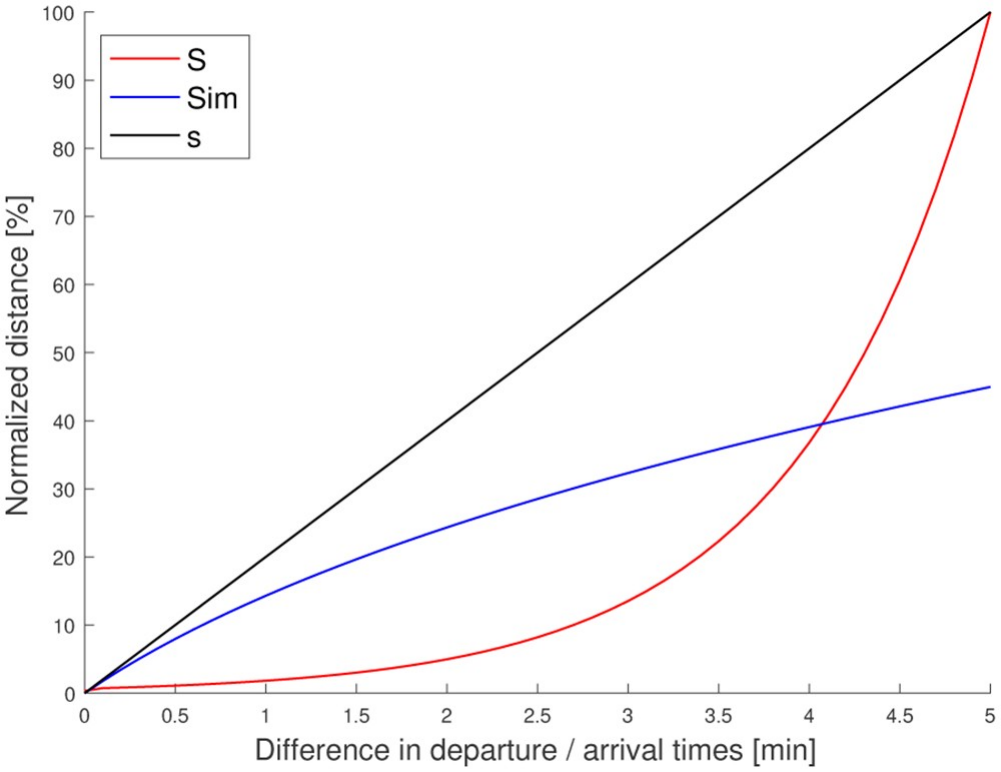
Comparison of different similarity indexes.

The main limitation of the approach described above to evaluate the similarity (such as the other attempts based on the Euclidean distance) is that it does not discriminate. It sufficiently enhances the difference between trips when applied to origin/destination locations and desired departure/arrival times only. Specifically, index of two trips with similar origin/destination, i.e. $d(p_{i}^{l},p_{j}^{l})$ close to zero (with *d* a distance function), but with significant difference in their desired departure/arrival times, i.e. high values of $|t_{i}^{l}-t_{j}^{l}|$, remains still low, see Fig 1. As a consequence, it deteriorates the ability of clustering methods to capture similar trips.

To overcome this drawback, we define a function *S*(*i*, *j*) to evaluate the similarity between two trips *i* and *j* based on the characteristics of their origin and destination [27]. From a physical point of view, the intuition is that two (or more) travelers may have the interest to share their trip if they start in the same neighborhood and at the same moment, and want to go to the same destination. The function *S* must encompass these different spatio-temporal attributes of the trips.

To fill this lack of modeling, we proposed the following function:

$$\begin{array}{c} \bar{S(i,j)}=\sum_{l\in [PU,DO]}\alpha_{l}e^{|f^{l}\left(i,j\right)|} \end{array}$$ (2)

where *f*^*l*^(*i*, *j*) is the feasibility function and *α*_*l*_ is a coefficient. Function *f* describes the service’s potential to operate the shared trips, i.e. the ability to pick up (or drop off) the two travelers before both of their desired departure times:

$$\begin{array}{c} f^{l}\left(i,j\right)=\left|t_{i}^{l}-t_{j}^{l}\right|-\gamma d\left(p_{i}^{l},p_{j}^{l}\right) \end{array}$$ (3)

where *γ* is the average pace to connect travelers that want to share a trip.

This parameter is a general and synthetic formula to describe the service’s operation and how it gathers two demand requests into the same vehicle: defining a meeting point, successive pick-ups, etc. For example, if the first traveler must walk to the second traveler’s pick-up point, then *γ* is equal to the inverse of the walking speed. If this distance is traveled by car, meaning that the service offers door-to-door service, then *γ* is equal to the inverse of the vehicle speed. Consequently, *f* is positive if the time difference between the two desired departure times $t_{i}^{PU}$ and $t_{j}^{PU}$ (or respectively the two arrival times) is enough to allow users to join. Whereas *f* is negative if travelers have to experience a delay in making the match possible.

Moreover, *α*_*l*_ is equal to $\frac{1}{2}$ if *f*^*l*^(*i*, *j*)>0 and to $\frac{3}{2}$ otherwise because it is more disadvantageous to be delayed.

In addition to this first index of similarity $\bar{S(i,j)}$, excessive distances/durations for rendezvous are penalized. Thus, penalties $\theta_{x}^{l}$ and $\theta_{t}^{l}$ are added when, respectively, the distances between origin (or destination) locations and departure (or arrival) times of trips *i* and *j* exceed, respectively, specific thresholds, $\delta_{x}^{l}$ and $\delta_{t}^{l}$:

$$\begin{array}{c} \theta_{x}^{l}=e^{d\left(p_{i}^{l},p_{j}^{l}\right)-\delta_{x}^{l}}\;\forall l\;/\;d\left(p_{i}^{l},p_{j}^{l}\right)>\delta_{x}^{l} \end{array}$$ (4)

$$\begin{array}{c} \theta_{t}^{l}=e^{|t_{i}^{l}-t_{j}^{l}|.\frac{\delta_{x}^{l}}{\delta_{t}^{l}}-\delta_{t}^{l}}\;\forall l\;/\;\left|t_{i}^{l},t_{j}^{l}\right|>\delta_{t}^{l} \end{array}$$ (5)

Otherwise, these penalties are null. In this manner, $S\left(i,j\right)=\bar{S(i,j)}+\theta_{x}^{l}+\theta_{t}^{l}$ defines a sharp function that enhances the differences between trips and facilitates identification of similar travelers in the data set. Notice that *S* is minimal (and equal to 1) when the two trips are identical.

Fig 1 highlights the evolution of *S* (in red) with regards to the difference between two hypothetical trips. A comparison with the similarity function *Sim* of [26] can be made. We complete this comparison by introducing a naive combination of the Euclidean distance and the absolute difference in time:

$$\begin{array}{c} s\left(i,j\right)=1+\sum_{l\in [PU,DO]}(|t_{i}^{l}-t_{j}^{l}|+d\left(p_{i}^{l},p_{j}^{l}\right)) \end{array}$$ (6)

The constant 1 is added to have the same minimal value than *S* and *Sim*. Notice that the different functions have been normalized to make them comparable. It is also important to remember that trips are similar when different functions are minimized. It turns out that *Sim* and *s* have a linear increase (quasi-linear for *Sim*) with the difference in departure/arrival times (curves of evolution with regard to the difference in origin/destination locations are strictly similar). On the opposite, our function *S* is much more discriminant. Consequently, differences between trips are clearly enhanced. This means that it will ease to cluster similar trips with good intra-cluster homogeneity and, simultaneously, a significant inter-cluster dissimilarity.

### Clustering similar trips

Now, we can identify groups of travelers likely to share a vehicle. To this end, a clustering method gathers the trips. Among the vast body of existing work, the main significant contributions for our study are the density-based methods. Indeed, we want to determine spatio-temporal areas where similar trips occur. Moreover, it is essential to use an algorithm to detect clusters of different densities, which is not the case for all density-based algorithms. Between the different choices, DB-SCAN holds our attention. This popular and classical method only requires two parameters: a threshold *ϵ* and a minimum number of points *MinPts*, which have to be in a radius *ϵ* so that the studied point is considered as an element of the cluster, see [28] for more details. The parameter *ϵ* is the maximal distance between trips, i.e. the maximal value of *S*, allowed to consider them as similar and group them into the same cluster. However, this method must be slightly adapted to detect groups of different density. We decide to perform successive DB-SCAN clustering, i.e. *itdbscan*, using the similarity function *S* as the distance while updating the parameter values iteratively. Starting with a large value of *MinPts* = *M* and a drastic *ϵ*, it makes it possible to identify large groups of travelers in the initial data set of trip *T*. In other terms, we first detect large and high-density clusters. Then, the DB-SCAN method is applied to the remaining non clustered trips to detect groups of minimal size *M* − 1. This process is repeated until *MinPts* = 2.

Simultaneously, we have defined an indicator of quality to select only the most promising clusters. A cluster can be considered satisfactory if locations of origin/destination and arrival/departure times of the trips within the cluster are relatively close. To this end, the function *S* is extended to consider sets of trips. In other terms, $|t_{i}^{l}-t_{j}^{l}|$ and $d(p_{i}^{l},p_{j}^{l})$ are respectively replaced by the mean distances, i.e. $\frac{1}{n_{k}}\sum_{i=1}^{n_{k}}\left|t_{i}^{l}-t_{j}^{l}\right|$ and $\frac{1}{n_{k}}\sum_{i=1}^{n_{k}}d\left(p_{i}^{l},p_{j}^{l}\right)$ where *n*_*k*_ is the number of trips inside the cluster *k*. We then normalized these values because the acceptable delays are strongly related to the length of the trips. Consequently, the quality index of cluster *k*, *Q*(*k*), is the function *S* applied to the set of clustered trips divided by the average length of the trips within-cluster *k*. Notice that we aim at minimizing the quality index, i.e. best clusters present values of *Q* close to zero. Indeed, *Q*(*k*) is low when (i) the spatio-temporal distance between origins is low, (ii) the spatio-temporal distance between destinations is low, and (iii) the mean travel distance is large. A cluster *k* is selected if and only if *Q*(*k*) is below a specific threshold *Q*_*max*_. In the remaining of the study, we set *Q*_*max*_ at 3. It corresponds to a restrictive matching policy.

Finally, Algorithm 1 *itdbscan* outlines the framework of the demand estimation method. Table 2 sums up the values of the parameters that are used to produce the results.

**Algorithm 1** Iterative DBSCAN—*itdbscan*

1: **function** *itdbscan* *data*, *similarityMatrix*, *minPtsMin*, *minPtsMax*, *epsMin*, *epsMax*, *Q*_*max*_

2:  mainClustering ←∅

3:  id ← 0

4:  test ← 0

5:  **for** minPts in {minPtsMax,minPtsMin} by step of -1 **do**

6:   **for** eps in {epsMin,epsMax} by step of 1 **do**

7:    clustering, K = dbscan(data,similarityMatrix,eps,minPts)

8:    **for** k in (0,K) by step of 1 **do**

9:     **if** Q(k) ≤ *Q*_*max*_ **then**

10:      Save k in mainClustering with the number id

11:      id ← id+1

12:      Delete travels of cluster k from data

13:      test ← 1

14:     **end if**

15:    **end for**

16:    **if** test == 1 **then**

17:     **if** data == ∅ **then**

18:      **break**

19:     **end if**

20:     Delete travels of cluster k from similarityMatrix

21:     test = 0

22:    **end if**

23:   **end for**

24:  **end for**

25:  **return** mainClustering

26: **end function**

**Table 2:** Values of the parameters used to produce the results.

Parameter	Value	Signification
*γ*	0.18 h/km	Average pace to connect travelers
$\delta_{t}^{PU}$	0.25 h	Threshold on departure times
$\delta_{t}^{DO}$	0.25 h	Threshold on arrival times
$\delta_{x}^{PU}$	0.25 km	Threshold on origin locations
$\delta_{x}^{DO}$	0.25 km	Threshold on destination locations
*Q*_*max*_	3	Maximal dissimilarity of clusters

## Results

### Detection of clusters of similar trips in New York City

**Fig 2:**
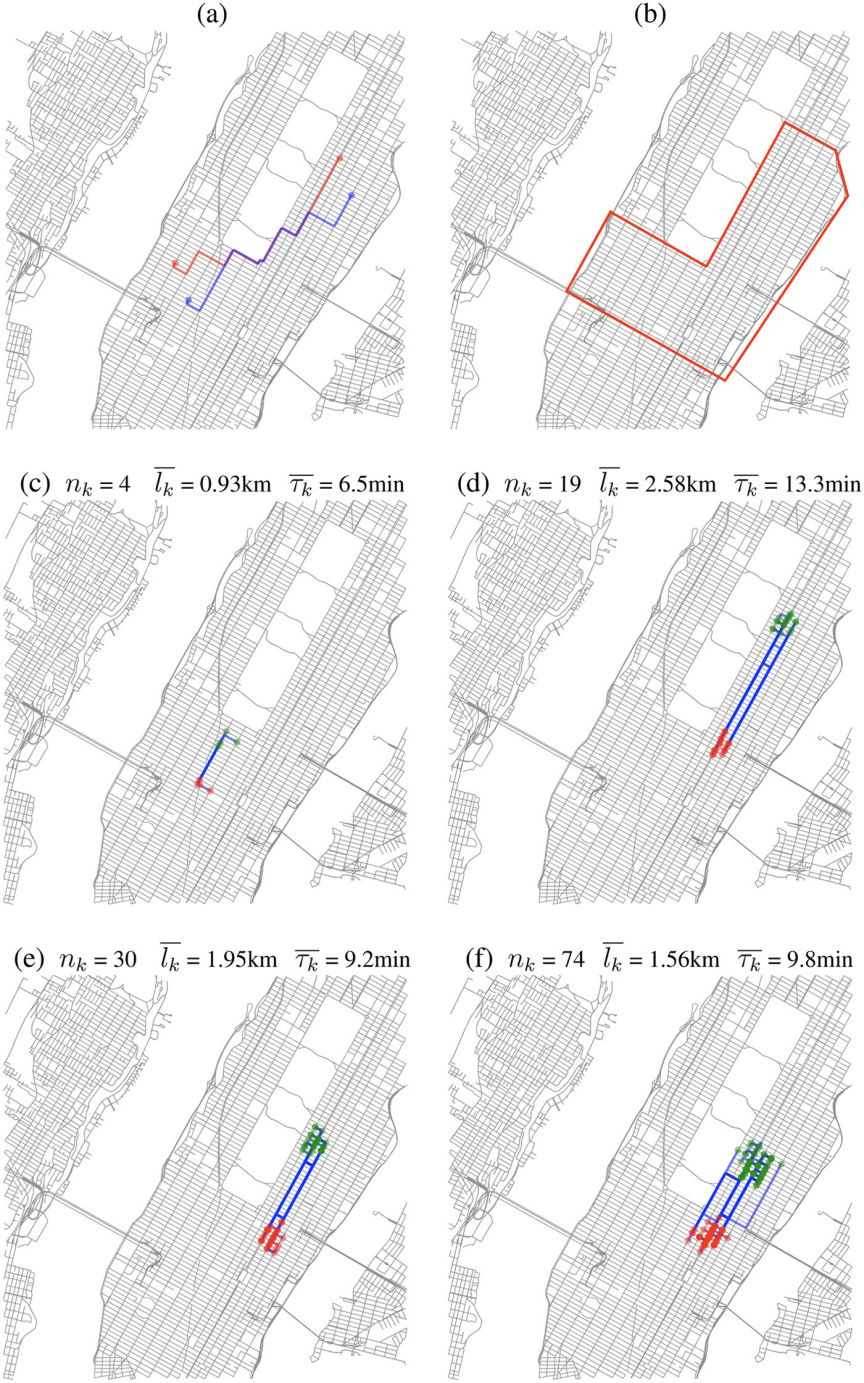
In the following, green markers denote departure points and red markers denote arrival points. Each point corresponds to a triplet (longitude, latitude, timestamp). (a) illustrates the functioning of the similarity function, departure and arrival points of each trips are compared in twos according to the feasibility function described in section Estimation of the shared mobility services’ demand—Modeling similarity between individual trips. (b) Depicts the area of study: Midtown and Upper East Side (Manhattan). (c), (d), (e), (f) shows clusters of different sizes and characteristics. *n*_*k*_ denotes the number of trips in the cluster *k*, $\bar{l_{k}}$ denotes the average length of trips in *k* and $\bar{\tau_{k}}$ denotes the average duration of the trips in *k*. Map data copyrighted OpenStreetMap contributors [29].

The clusters are firstly estimated for a random test-day. The visual analysis of the trips, projected on the road-map network, of four of these clusters Fig 2 reveals that the origin/destination whereabouts are adjacent and the differences in departure/arrival times remain low. Furthermore, clusters have different sizes, from 2 trips to 74 trips gathered into the same group. It brings to light that the shared mobility demand may take many aspects requiring different forms of transportation services to be optimally satisfied. To go further on the analysis, average distances between origin and respectively destination locations within cluster *k* are defined $\bar{D_{k}^{o}}=\frac{\sum_{i=1}^{n_{k}-1}\sum_{j=i+1}^{n_{k}}D\left(o_{i},o_{j}\right)}{\frac{1}{2}n_{k}.\left(n_{k}-1\right)}$ and $\bar{D_{k}^{d}}=\frac{\sum_{i=1}^{n_{k}-1}\sum_{j=i+1}^{n_{k}}D\left(d_{i},d_{j}\right)}{\frac{1}{2}n_{k}.\left(n_{k}-1\right)}$ where *n*_*k*_ is the size of the cluster *k* and *D* is the Euclidean distance. Similarly, average offsets between departure / arrival times are given by $\bar{T_{k}^{t}}=\frac{\sum_{i=1}^{n_{k}-1}\sum_{j=i+1}^{n_{k}}\left|t_{i}-t_{j}\right|}{\frac{1}{2}n_{k}.\left(n_{k}-1\right)}$ and $\bar{T_{k}^{a}}=\frac{\sum_{i=1}^{n_{k}-1}\sum_{j=i+1}^{n_{k}}\left|a_{i}-a_{j}\right|}{\frac{1}{2}n_{k}.\left(n_{k}-1\right)}$. Moreover, the average travel length $\bar{l_{k}}$ is directly the arithmetic average of the length of *n*_*k*_ trips within the cluster *k*, whereas the average travel time $\bar{\tau_{k}}$ is the arithmetic average of the duration of the *n*_*k*_ trips.

Interestingly, the features of these 4 clusters reveal that the trips that have been gathered for each of them are similar. Origins/destinations are located in the same area of the city (the average distance between locations is 220 m). The average offsets in the departure/arrival times are also very similar (the average offsets between departure and respectively arrival times are 12.5 minutes and 13.5 minutes). We focus our study on the long-term human mobility and not on real-time request satisfaction. The delay may become tolerable because it can be balanced by a change in departure time and the benefits of sharing vehicles such as cost reduction, positive action for the environment, and social interaction. The regularity in the day-to-day pattern is a point determinant to favor a modal shift from a single-occupancy vehicle to a shared mobility system.

**Fig 3:**
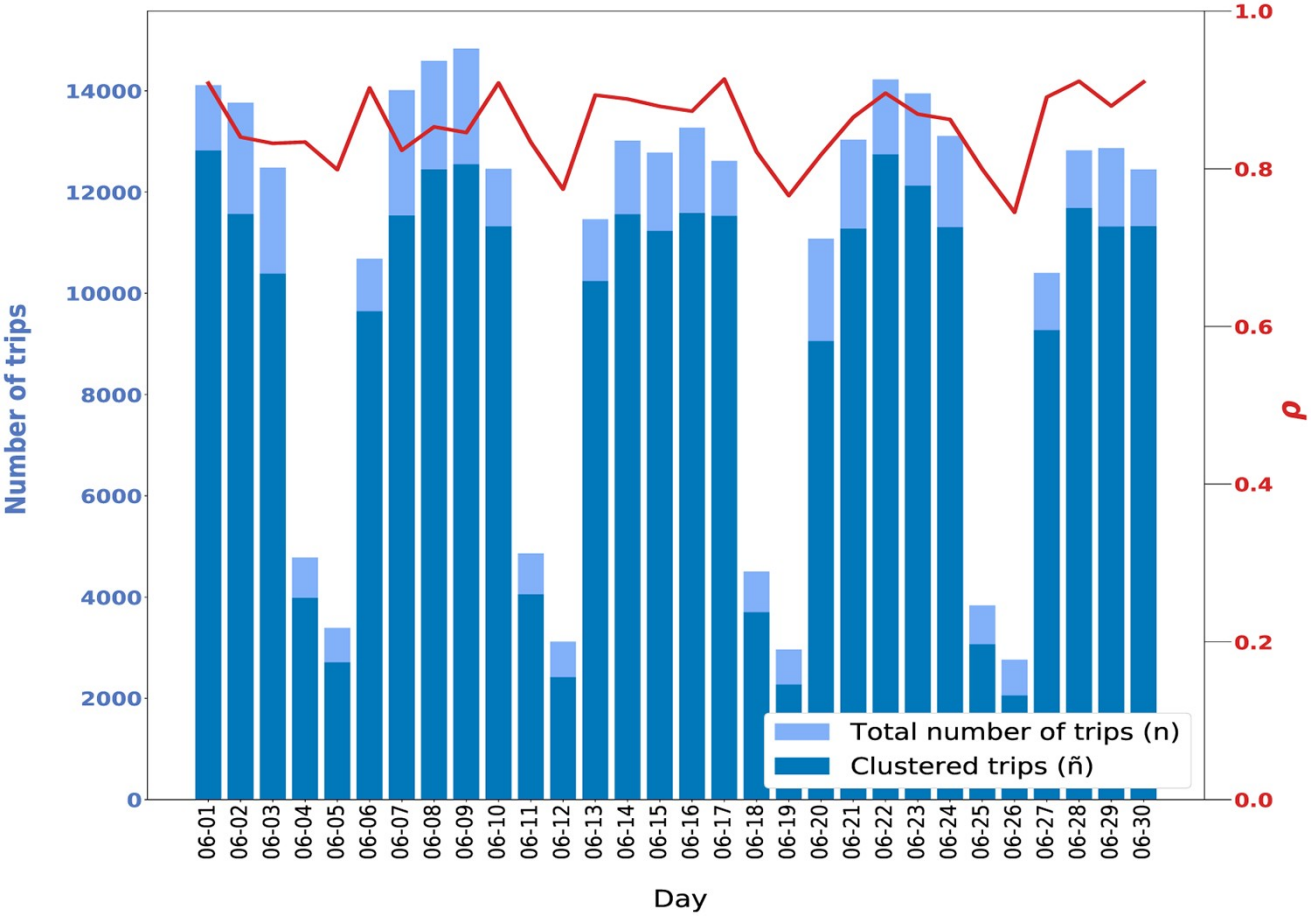
Number of clustered trips per day in New York City (Midtown and Upper East Side) from 8h to 11h. The ratio *ρ* of the clustered trips over the total trips is very constant for one weekday to the other. The recurrent variations correspond to week-ends.

The study can now be extended to the whole dataset. The quality of the clustering results is assessed through three indicators: (i) $\tilde{n}$ the total number of trips that have been gathered, (ii) the ratio *ρ* of trips that have been gathered with *n* the total number of trips within the period, and (iii) the number of estimated clusters *K*. Fig 3 depicts the number of trips clustered by day and the ratio *ρ*. We observe that, on average, each day more than 85% of trips are clustered according to their spatio-temporal commonalities. This clustering generated 19.466 clusters in which all the clustered trips of the 14 days are distributed. This result means that each day, on average, from 8h to 11h, we detect more than 1300 spatio-temporal areas containing similar trips.

Because the characteristics of the clusters are remarkably stable among the observation days, we investigate if commonalities in the clusters can be identified. Many approaches exist to derive the most representative partition from a group of partitions, such as meta-clustering or consensus cluster method [7]. Here, we use the same clustering method to maintain consistency when scaling-up. Thus, for each of the 19.466 clusters identified for 14 days of the dataset, centroids are calculated. Centroids correspond to the mean origin/destination locations and mean departure/arrival times of the clustered trips. This information can be handy to design the transportation supply because centroids can be the location of common meeting points of the standby areas of shared vehicles.

**Fig 4:**
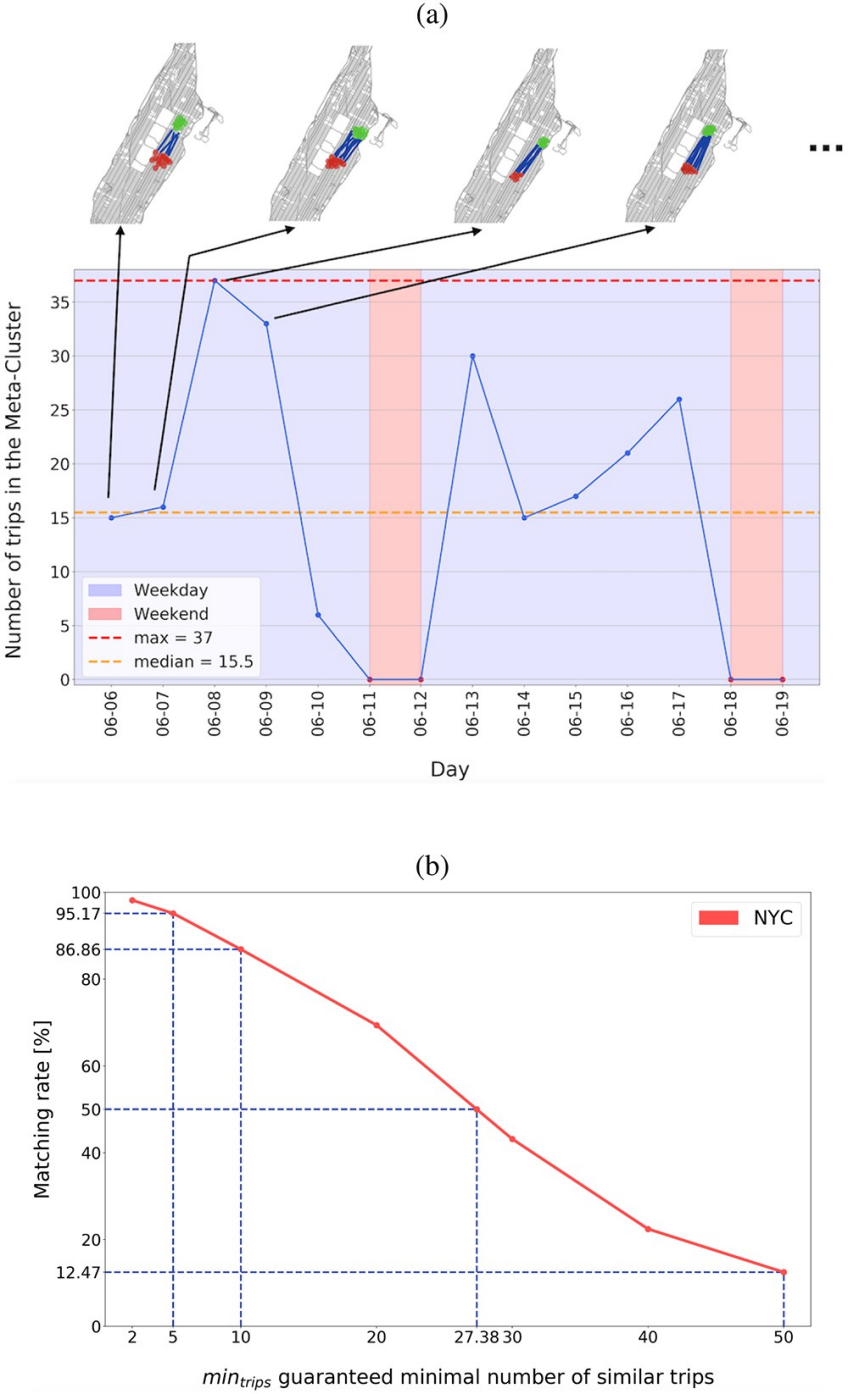
(a) Example of meta-cluster: evolution of the number of trips that can be gathered each day. Effects of week-end clearly appears. During weekdays, daily clusters size is almost always bigger than 15 trips. Meanwhile, the inspection of the locations of the 4 first daily clusters confirms the similarity in the selected trips (figures on the top). For this specific case, it can be easily imagined that this demand could have been served by a small van or an on-demand transit system. (b) Evolution of the ratio of future trips that can be clustered in groups of size *min*_*trips*_. The value of *min*_*trips*_ is minimal number of similar trips that can be guaranteed. © OpenStreetMap Contributor.

Indexes of similarity *S* between any pairs of centroids of the 19.466 clusters are calculated. Then, centroids are gathered to obtain clusters of groups of similar trips, i.e. meta-clusters. Interestingly, more than 94% of the daily clusters are recurrent from one day to the other. The most interesting point is to observe the daily trips forming a randomly selected meta-cluster. Simultaneous analysis of the evolution of the size of the daily cluster and the localization of the related origin/destination whereabouts (Fig 4a) shows that similar trips can be observed every day, except on weekends. It is important to note that different individuals perform these trips from one day to another. However, global human mobility is remarkably regular; this is a valuable insight to efficiently tune transportation services and favor shared mobility. For the specific case highlighted in Fig 4, the estimated demand can be served by dispatching, every morning, small vans (or customized buses) in the vicinity of the origin whereabouts and then use these vehicles later during the day for other groups of trips. For the whole set of the meta-clusters, the average length of a trip is around 2.7 km (road distance), and the average travel time is about 11.1 min. Table 3 shows the results for the whole set of meta-clusters. The characteristics of these meta-clusters, are very similar from those observed for the daily clusters. It also confirms the robusteness of our demand estimation method.

**Table 3:** Metrics of the meta-clusters estimated from the daily clusters of the period between June 06, 2011 and the June 19, 2011.

Description	Value
Number of Meta-clusters	2136
Number of clusters grouped to form Meta-clusters	18456
Mean number of clusters by Meta-cluster	8.64
Mean distance between origins and the origins’ centroids	0.2 km
Mean distance between destinations and the destinations’ centroids	0.2 km
Mean offset time between origins and the origins’ centroids	6.16 min
Mean offset time between destinations and the destinations’ centroids	6.27 min
Mean distance of travels	2.7 km
Mean travels time	11.1 min

### Projected model

We are now going to take advantage of the above significant result and verify that the identification of the regular pattern can be used to gather individual trips into the same vehicle. In other words, we want to investigate that the estimated meta-clusters can predict the number of similar trips for a future traveler and thus anticipate the shared mobility service demand, as claimed before.

The first step is to determine if a trip *T* of new observation day can be included in a meta-cluster without degrading the quality (according to the values of similarity defined in Table 2). Let *M* be a meta-cluster and $T_{a}^{d}$ the hypothetical trip described by the centroids of the departures and arrivals of *M*. A trip *T* of a new observation day is included in a meta-cluster *M* if the similarity between $T_{a}^{d}$ and *T* is lower than the maximal similarity between any trip of the meta-cluster *M* and $T_{a}^{d}$. It means that a traveler will have a high probability of finding at least another user performing a similar trip.

Secondly, since the inclusion in a meta-cluster must guarantee a minimum number of similar trips, this condition must be verified to confirm the predictive power of the global methodology. Consequently, the minimum number *min*_*trips*_ of trips that can be included in the meta-cluster *M* is determined. This condition means that if a new trip *T* is in a meta-cluster, then there will be at least *min*_*trips*_ in M similar to *T* (according to the values of similarity defined in Table 2). In other words, for a new random trip at a given hour and location, it estimates the minimal number of travelers performing a similar trip. Fig 4b depicts the percentage of trips for a new day that can be included into a meta-cluster in function of the value of *min*_*trips*_.

In the New York City dataset, it can be observed that more than 12% of the realized trips could have been gathered in groups of 50 travelers. It means that massive shared mobility systems, such as transit on-demand services, can find potential customers and yield to a significant reduction of the number of cars flowing in the city. Interestingly, every weekday, one traveler over two might find at least 27 other users to share a vehicle with an acceptable delay. Shared mobility systems with smaller capacity (such as ridesharing, carpooling, small van, or future shared autonomous vehicles services) could also find their appropriate demand [30]. Indeed, more than 85% of the trips can be clustered in groups of size smaller than 10. If only pairs of similar trips are searched, this value increases until 98%, a result close to the one proposed by [16]. Notice that this very high matching rate is due to the high density of trips in the studied area. However, almost as good results are obtained for a less dense city (see S1 Text). To the best of our knowledge, such a method does not exist in the literature. It allows estimating for any future trips in a city at a given time the potential number of passengers who can perform a similar trip. Moreover, these results confirm that a significant ratio of trips could be grouped into the same vehicle every day.

## Conclusion

In this paper, we investigated whether groups of travelers that realize similar trips in a city exist. These users constitute the potential demand for future shared mobility services such as ride-sharing, shared taxis, or carpooling. The ability to share a trip with another traveler relies on factors such as cost, comfort, reliability, etc. To tackle this challenge, only spatio-temporal features are considered here: origin and destination, desired departure, and arrival times. Based on these attributes, a general methodology has been elaborated and experimented using data giving access to this information: the set of trips realized by users of taxi and limousine services in NYC.

The methodology mainly relies on a function to evaluate the similarity between any pair of trips. This index drastically differs from the existing works because the shape of the function is very sharp (making it particularly convenient for clustering) and considers the feasibility of the trip sharing, i.e. ability of the transportation service to realize multiple pick-up/drop-off. Then, the classical DB-SCAN clustering method has been adapted to the specific case of the shared mobility services demand estimation. We have designed an algorithm able to gather similar trips into groups of different sizes and characteristics by fixing only the maximum dissimilarity allowed between trips of the same group. This method has then been experimented using a data set released by the New York City Taxi and Limousine Commission. However, the method is enough general to be performed with any other source of data giving access to origin/destination and desired departure/arrival times of trips: GPS trajectories, OD matrices, travel surveys, etc.

The results show that more than 80% of the trips can be clustered for the 30 observation days of the data set by maintaining a relatively restrictive matching policy. Travelers are gathered on groups of different sizes: from pairs of travelers to groups of 70 users that realize almost the same trips at the same time. The most significant part of the clusters is composed of groups of 2 or 3 travelers, meaning that services such as ride-sharing, shared taxis, or carpooling are the most promising solutions between all the different versions of shared mobility services. Interestingly, characteristics of the clustered trips are entirely consistent with the fundamental idea of what similar trips should be: the average length is around 2.7km (road distance), whereas the average travel time is about 11 min. The travelers wishing to share their trips must bring them back or forward 6 minutes on average, which is entirely acceptable.

Finally, we start to investigate if commonalities exist between the clusters of 14 successive days. Using the same clustering method, regular patterns have been identified among the daily clusters, i.e. meta-clusters. Specifically, large clusters are recurrent, i.e. one can observe a significant number of trips with the same origin/destination and desired departure/arrival times from one day to the other. The analysis of meta-clusters shows that 94% of the trips are recurrent. Detection of regular patterns is a point essential because it ensures the presence of the potential daily users for the transportation network companies. For individuals, the transportation supply must be stable over time; otherwise, private vehicle continues to be the best option. Thus, this stability of the demand pattern ensures that the critical mass is reached every day to enable shared trip options. These options can be provided either by the individuals themselves (ride-sharing such as carpooling or vanpooling) or by transportation companies (ride-sourcing such as shared taxi or shuttle). As indicated in S1 File, our method is generic and reproducible with datasets coming from different cities in the world such as New York City, US, and Chengdu, China, in this paper; Lyon, France, is under investigation. In conclusion, the method presented in this paper provides a valued design pattern for the implementation of new shared mobility services. These elements suggest that shared autonomous vehicle solutions may be viable, from a pure transport perspective.

## Supporting information

S1 File (ZIP)

## References

[1] Song C, Qu Z, Blumm N, Barabási AL. Limits of predictability in human mobility. Science. 2010;327(5968):1018–1021. 10.1126/science.1177170 20167789

[2] Alessandretti L, Sapiezynski P, Sekara V, Lehmann S, Baronchelli A. Evidence for a conserved quantity in human mobility. Nature Human Behaviour. 2018;2(7):485–491. 10.1038/s41562-018-0364-x 31097800

[3] Giannotti F, Nanni M, Pedreschi D, Pinelli F, Renso C, Rinzivillo S, et al Unveiling the complexity of human mobility by querying and mining massive trajectory data. The VLDB Journal. 2011;20(5):695 10.1007/s00778-011-0244-8

[4] Song C, Koren T, Wang P, Barabási AL. Modelling the scaling properties of human mobility. Nature Physics. 2010;6(10):818–823. 10.1038/nphys1760

[5] Gonzalez MC, Hidalgo CA, Barabasi AL. Understanding individual human mobility patterns. nature. 2008;453(7196):779–782. 10.1038/nature06958 18528393

[6] Wang XW, Han XP, Wang BH. Correlations and scaling laws in human mobility. PloS one. 2014;9(1). 10.1371/journal.pone.0084954 PMC3890294 24454769

[7] Lopez C, Leclercq L, Krishnakumari P, Chiabaut N, Van Lint H. Revealing the day-to-day regularity of urban congestion patterns with 3D speed maps. Scientific Reports. 2017;7(1):14029 10.1038/s41598-017-14237-8 29070859 PMC5656590

[8] Wang P, Hunter T, Bayen AM, Schechtner K, González MC. Understanding road usage patterns in urban areas. Scientific reports. 2012;2:1001 10.1038/srep01001 23259045 PMC3526957

[9] Akbarzadeh M, Estrada E. Communicability geometry captures traffic flows in cities. Nature Human Behaviour. 2018;2(9):645–652. 10.1038/s41562-018-0407-3 31346275

[10] Zhao K, Musolesi M, Hui P, Rao W, Tarkoma S. Explaining the power-law distribution of human mobility through transportationmodality decomposition. Scientific reports. 2015;5(1):1–7. 10.1038/srep09136 PMC5375979 25779306

[11] Erhardt GD, Roy S, Cooper D, Sana B, Chen M, Castiglione J. Do transportation network companies decrease or increase congestion? Science advances. 2019;5(5):eaau2670 10.1126/sciadv.aau2670 31086811 PMC6506243

[12] Rayle L, Dai D, Chan N, Cervero R, Shaheen S. Just a better taxi? A survey-based comparison of taxis, transit, and ridesourcing services in San Francisco. Transport Policy. 2016;45:168–178. 10.1016/j.tranpol.2015.10.004

[13] Furuhata M, Dessouky M, Ordóñez F, Brunet ME, Wang X, Koenig S. Ridesharing: The state of the art and future directions. Transportation Research Part B: Methodological. 2013;57:28–46. 10.1016/j.trb.2013.08.012

[14] Agatz N, Erera A, Savelsbergh M, Wang X. Optimization for dynamic ride-sharing: A review. European Journal of Operational Research. 2012;223(2):295–303. 10.1016/j.ejor.2012.05.028

[15] Ray JB. Planning a real-time ridesharing network: critical mass and role of transfers. In: Transport Research Arena (TRA) 5th Conference; 2014.

[16] Santi P, Resta G, Szell M, Sobolevsky S, Strogatz SH, Ratti C. Quantifying the benefits of vehicle pooling with shareability networks. Proceedings of the National Academy of Sciences. 2014;111(37):13290–13294. 10.1073/pnas.1403657111 PMC4169909 25197046

[17] Tachet R, Sagarra O, Santi P, Resta G, Szell M, Strogatz S, et al Scaling law of urban ride sharing. Scientific reports. 2017;7:42868 10.1038/srep42868 28262743 PMC5337932

[18] Ramírez HG, Leclercq L, Chiabaut N, Becarie C, Krug J. Unravelling travellers’ route choice behaviour at full-scale urban network by focusing on representative OD pairs in computer experiments. PloS one. 2019;14(11). 10.1371/journal.pone.0225069 PMC6850682 31714945

[19] Creutzig F, Jochem P, Edelenbosch OY, Mattauch L, van Vuuren DP, McCollum D, et al Transport: A roadblock to climate change mitigation? Science. 2015;350(6263):911–912. 10.1126/science.aac8033 26586747

[20] NYC Department of Transportation. New York City Mobility Report; 2019. https://www1.nyc.gov/html/dot/downloads/pdf/mobility-report-singlepage-2019.pdf.

[21] Abraham S, Lal PS. Spatio-temporal similarity of network-constrained moving object trajectories using sequence alignment of travel locations. Transportation research part C: emerging technologies. 2012;23:109–123. 10.1016/j.trc.2011.12.008

[22] Tiakas E, Papadopoulos AN, Nanopoulos A, Manolopoulos Y, Stojanovic D, Djordjevic-Kajan S. Trajectory similarity search in spatial networks. In: 2006 10th International Database Engineering and Applications Symposium (IDEAS’06). IEEE; 2006. p. 185–192.

[23] Toohey K, Duckham M. Trajectory similarity measures. Sigspatial Special. 2015;7(1):43–50. 10.1145/2782759.2782767

[24] van Kreveld M, Luo J. The definition and computation of trajectory and subtrajectory similarity. In: Proceedings of the 15th annual ACM international symposium on Advances in geographic information systems. ACM; 2007. p. 44.

[25] Wang H, Su H, Zheng K, Sadiq S, Zhou X. An effectiveness study on trajectory similarity measures. In: Proceedings of the Twenty-Fourth Australasian Database Conference-Volume 137. Australian Computer Society, Inc.; 2013. p. 13–22.

[26] Ketabi R, Alipour B, Helmy A. Playing with matches: vehicular mobility through analysis of trip similarity and matching. In: Proceedings of the 26th ACM SIGSPATIAL International Conference on Advances in Geographic Information Systems; 2018. p. 544–547.

[27] Chiabaut N, Veve C. Identifying Twin Travelers Using Ridesourcing Trip Data. Transport Findings. 2019.

[28] Ester M, Kriegel HP, Sander J, Xu X, et al A density-based algorithm for discovering clusters in large spatial databases with noise. In: Kdd. vol. 96; 1996 p. 226–231.

[29] contributors O. Planet dump retrieved from https://planet.osm.org; 2017. https://www.openstreetmap.org.

[30] Duarte F. Self-driving cars: A city perspective. Science Robotics. 2019;4(28):eaav9843 10.1126/scirobotics.aav9843 33137749

