# Supplementary material for: Estimation of the shared mobility demand based on the daily regularity of the urban mobility and the similarity of individual trips
Source: PLoS One. 2020 Sep 17;15(9):e0238143. doi: 10.1371/journal.pone.0238143 (PMC7497992; doi:10.1371/journal.pone.0238143)
Supplement: S1 File — (ZIP) [file pone.0238143.s001.zip › Supplementary Material.pdf]

Supplementary Material

VEVE Cyril[1], CHIABAUT Nicolas[1,*]

[1] Univ. Lyon, Univ. Gustave Eiffel, ENTPE, LICIT, F-69518 Vaulx-En-Velin, France

[*]Corresponding author : nicolas.chiabaut@entpe.fr

Abstract

Even if shared mobility services are encouraged by transportation policies, they remain underused and inefficient transportation modes because they struggle to find their customer base. This paper aims to estimate the potential demand for such services by focusing on individual trips and determining the number of passengers who perform similar trips. Contrary to existing papers, this study focuses on the demand without assuming any specific shared mobility system. The experiment performed on data coming from New York City conducts to cluster more than 85% of the trips. Consequently, shared mobility services such as ride-sharing can find their customer base and, at a long time, to a significantly reduce the number of cars flowing in the city. After a detailed analysis, commonalities in the clusters are identified: regular patterns from one day to the next exist in shared mobility demand. This regularity makes it possible to anticipate the potential shared mobility demand to help transportation suppliers to optimize their operations.

S1 Case of study in Chengdu - CHINA

The method presented in the article has been also performed on a dataset provided by the most important Transportation Network Company in China: Didi Chuxing (data source: https://outreach.didichuxing.com. This company has released two months of data consisting of more than 6 millions trips performed by their drivers [1]. To compare these results with those performed in New York City, the study has been focused on morning peak hours from 8h to 11h. For each trip i, this data set gives access to the following information: departure time t_i^{PU} and location $p_i^{PU} = (x_i^{PU}, y_i^{PU})$ of the pick-up of the passenger(s); and arrival time t_i^{DO} and location $p_i^{DO} = (x_i^{DO}, y_i^{DO})$ of the drop-off. Moreover, we consider that these observations correspond to the desired departure/arrival times and origins/destinations of the travelers. Notice that the Chinese government imposes restriction on the use of geographical data. Consequently, random offsets on positions are added to generate a specific coordinate system (GCJ-02). However, reverse transformations are available to convert data from GCJ-02 to WGS84.

The used variables for the function of similarity and the clustering algorithm are the same than those used in New York City (Section Estimation of the shared mobility services' demand - Table 1), with the exception of Q_{max}. Indeed, because of the different sizes of the studied areas, we set $Q_{max} = 1.7$ for this dataset. The origin o_i and destination d_i whereabouts, and the departure t_i and arrival a_i times of more than 450.000 trips are recorded in this area.

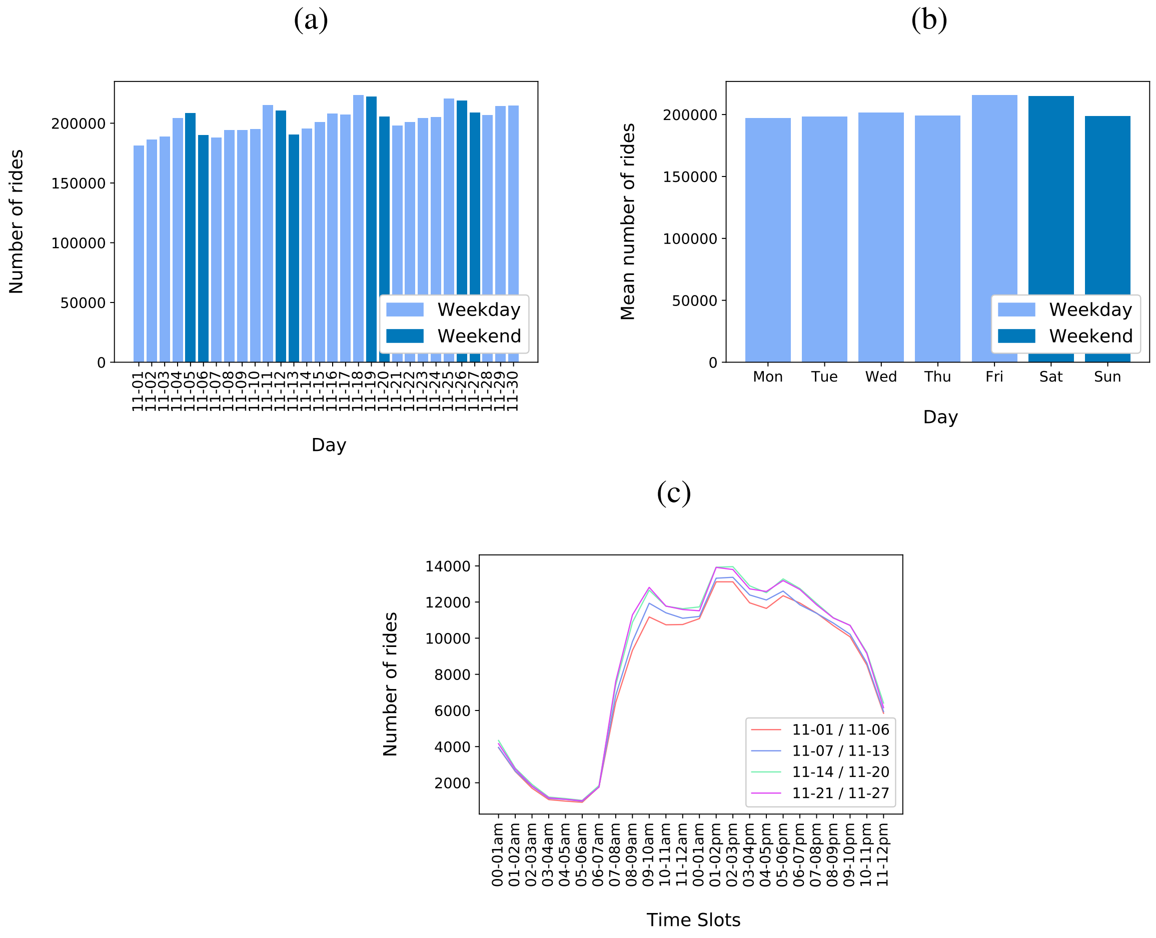

Fig S1.1. (a) Number of rides per day; (b) Mean number of rides per day; (c) Weekly mean number of rides per hour (Didi Chuxing Data collected in Chengdu, China, in November 2016);

Figure S1.1.a shows the distribution of the number of trips per day and reveals that the number of travelers served by Didi's drivers is relatively constant (between 180.000 and 225.000 rides per day). Figure S1.1.b presents the average number of trips for each type of day. It appears that Friday and Saturday are the days where the demand is highest. To complete these observations, Figure S1.1.c depicts the variations of the number of trips with the time of the day. It turns out that four peak periods (or with a number of trips higher than the rest of the day) can be identified: 8h to 10h, 13h to 15h, and 17h to 19h. However, there is no significant variation between 6h to 20h. The study focused only on trips located in a restricted zone, representing a circle of 5.5 km radius and centered on the Sichuan Science and Technology Museum.

In the same way, as in New York City, the 14 days meta-clustering performed from November, 14th to 27th, 2016 detected a large number of meta-clusters. An example of meta-cluster in Chengdu is depicted in Figure S1.2. Table S1.1 shows the results for the whole set of meta-clusters. We observe that the number of meta-clusters found is greater than in New York City, but this number represents only 73% of the initial number of clusters found. In other words, in Chengdu 73% of clusters initially found are recurrent day to day, while in the studied area in New York 94% of clusters initially found are recurrent. It seems that this number is correlated with the density of trips in the studied area. Moreover, mean travel times and distances are higher than in New York. This result appears as normal, given that the studied area

in Chengdu is more extensive than in New-York City.

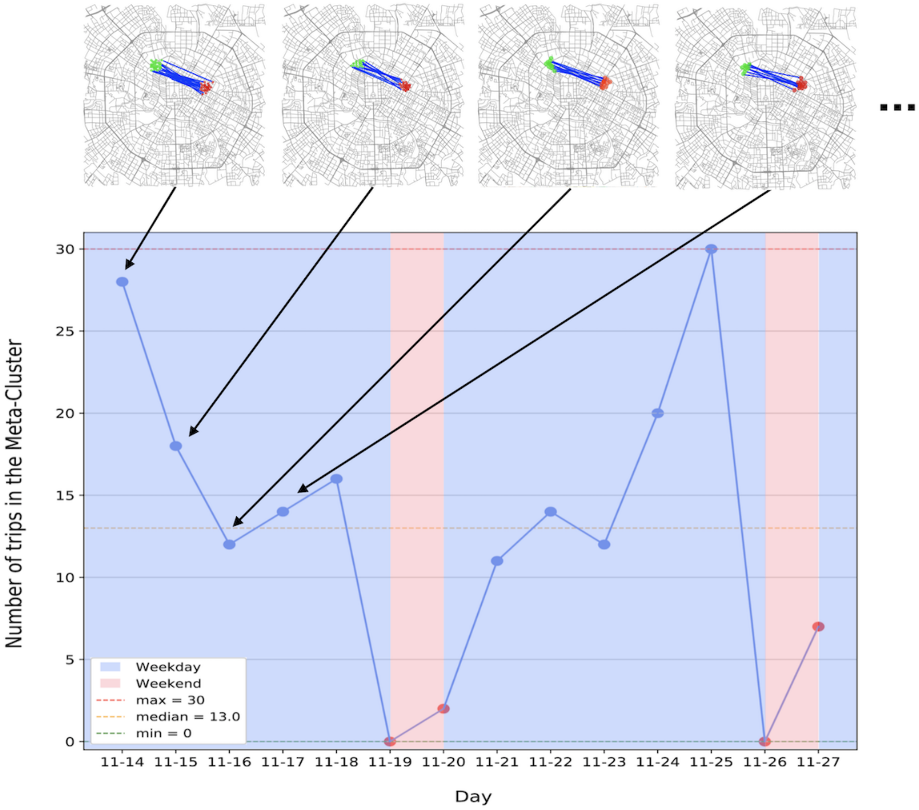

Fig S1.2. Analysis of the daily trips forming a meta-cluster: similar trips can be observed every day

Description	Value
Number of Meta-clusters	6301
Mean distance between origins	0.5 km
Mean distance between destinations	0.48 km
Mean offset time between origins	10.8 min
Mean offset time between destinations	11.0 min
Mean distance of travels	4.41 km
Mean travel times	19.0 min

Table S1.1. Metrics of the meta-clusters estimated from the daily clusters of the period between November 14, 2016 and the November 27, 2016 © OpenStreetMap Contributors

For a future given trip T, the estimation of the minimal number of travelers performing similar trips is lower than in New York City. Even if more than 20% of the realized trips could have been gathered with pairs of other trips, and 8.13% could have been gathered in groups of 5 travelers. These results are not comparable to those in New York City. Indeed, the values of min_{trips} for which we can have enough matching rate are smaller than those in New York. In other words, in more than 20% of cases, we can find a pair of travelers who perform a similar trip. Whereas with the data collected in New York in a dense area, the matching rate is close to 100%. Moreover, the values for which there is a consistent matching rate are greater with the

New York taxis data. Therefore, we can suppose that there exists a link between the density of trips in the observed area and a high matching rate for high values of min_{trips}.

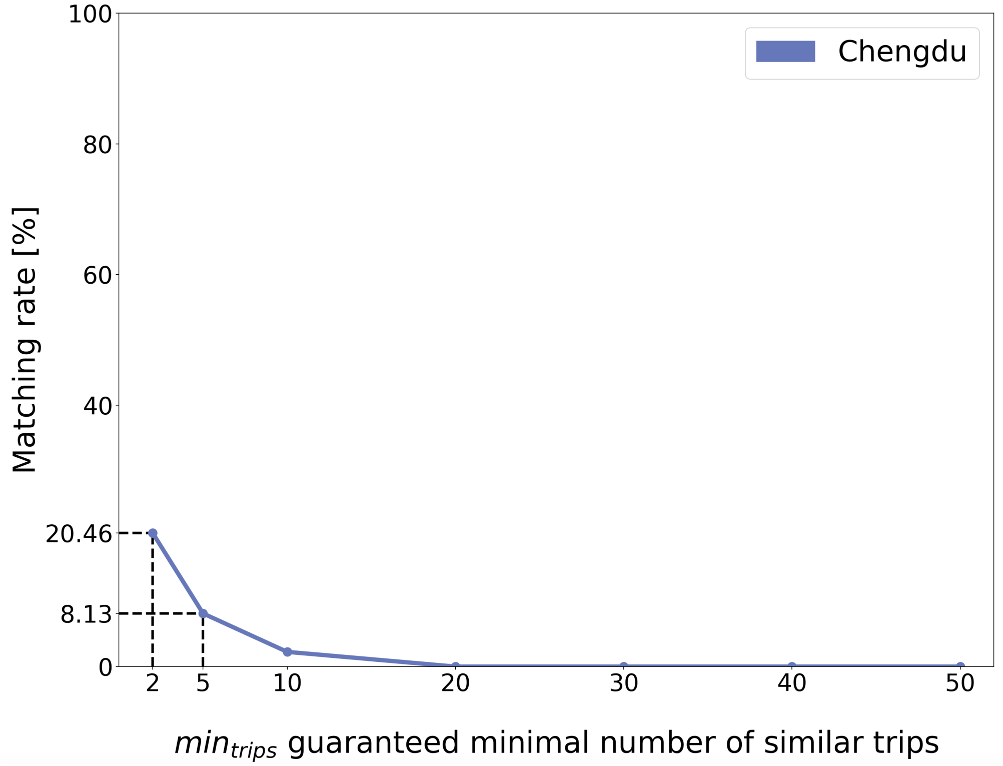

Fig S1.3. Evolution of the ratio of future trips that can be clustered in groups of size min_{trips} in Chengdu.

References

1. Xu C, Gao J, Zuo F, Ozbay K, Yang H, Cui H. Understanding Spatial-Temporal Impacts on Mode Preference between Taxi and E-Hailing Service. 98th Annual Meeting of the Transportation Research Board. 2019;(19-05001).
